# Supplementary material for: Collaborative intelligence in AI: Evaluating the performance of a council of AIs on the USMLE
Source: PLOS Digit Health. 2025 Oct 9;4(10):e0000787. doi: 10.1371/journal.pdig.0000787 (PMC12510544; doi:10.1371/journal.pdig.0000787)
Supplement: S2 File — (ZIP) [file pdig.0000787.s002.zip › S2 Data Raw data files containing unprocessed question inputs and LLM outputs including transcripts of deliberations/STEP3_Question-36-1_Type-MC-NJ.docx]

**Facilitator** **[Round 1]:** A 15-year-old girl is brought to the office by her parents because of a 2-month history of progressively worsening gait disturbance. She has fallen several times, once striking her head and causing a brief loss of consciousness. Her parents state that she has had behavioral changes during this time. Medical history is remarkable for asthma treated with a corticosteroid inhaler as needed. Family history is remarkable for multiple sclerosis in a maternal aunt, parkinsonism in her paternal grandfather, and cancer in several paternal relatives, including a grandmother who died from a hemangioblastoma. Vital signs are normal. The patient is awake and alert, but she avoids eye contact and will not answer questions. Auscultation of the lungs discloses expiratory wheezing. Cardiac and abdominal examinations disclose no abnormalities. She has mild bilateral papilledema. Her stance and gait are wide-based, and she is unable to perform a tandem walk. Toxicology screening of the urine is positive for marijuana and cocaine metabolites. Which of the following is the most likely initial working diagnosis? (A) Cerebellar tumor (B) Drug-related ataxia (C) Epidural hematoma (D) Juvenile-onset parkinsonism (E) Multiple sclerosis

**AI_E:** RESTATING THE QUESTION: This case involves a young teenager who is demonstrating a progressively worsening gait disturbance, has shown behavioral modifications, and also has a medical history of asthma. Her family history presents some neurological disorders (multiple sclerosis and parkinsonism) and cancers, including a hemangioblastoma (a tumor formed in blood vessels usually appearing in the cerebellum, spinal cord, or retina). There are no abnormal signs from her physical examination, except for bilateral papilledema (optic disc swelling usually caused by increased intracranial pressure) and abnormal gait. She is also found to be using marijuana and cocaine. The question is: What's the most likely initial diagnosis to investigate further given these findings? PLANNING: A few assumptions including findings, signs and symptoms, and patient history can lead us to different diagnoses. However, taking into consideration the rapid two-month progression of gait disturbance and behavioral changes, bilateral papilledema, family history of hemangioblastoma, and her age; leads us to hypothesize the presence of a CNS tumor, likely cerebellar, as the primary clinical suspicion. CLINICAL REASONING: While drug-related ataxia can be considered given the positive toxicology screening, it doesn't account for the presence of bilateral papilledema, a telltale sign of increased intracranial pressure caused by an intracranial space-occupying lesion, such as a tumor or hematoma. Epidural hematoma is usually acute and followed by a significant trauma, which isn't reported here. Juvenile-onset parkinsonism, generally presents with stiffness, resting tremor, and bradykinesia but not typically with papilledema. While family history should be considered, multiple sclerosis usually manifests as remitting-relapsing neurologic deficits separated by time and space, often without papilledema. CONCLUDING: Given that the patient’s presentation includes rapid progression of neurological symptoms, a wide-based stance and gait, behavioral changes, and bilateral papilledema, all these indications lead us to suspect an intracranial space-occupying lesion. In light of these details and the family history, a cerebellar tumor seems like a plausible first diagnosis to consider for further investigation. RESPONSE: Hence, the most likely working diagnosis in this case would be (A) Cerebellar tumor.

**AI_B:** 1) RESTATING THE QUESTION: The presented case features a 15-year-old girl, who has been displaying an unsteady walk or gait disturbance for the last two months and has experienced episodic falls including an episode of brief loss of consciousness after hitting her head. There are perceptible changes in her behavior according to her parents. Her past medical history discloses asthma managed with a corticosteroid inhaler. Significant family history comprises multiple sclerosis in the maternal aunt, parkinsonism in paternal grandfather, and cancer, including a grandmother with hemangioblastoma. Physical examination findings, such as diminished interaction, mild bilateral papilledema, and wide-based unsteady gait, are noted. Drugs, namely marijuana and cocaine, have been detected in her urine. 2) PLANNING: The options available for the initial working diagnosis include cerebellar tumor, drug-related ataxia, epidural hematoma, juvenile-onset parkinsonism, and multiple sclerosis. These options are formulated considering her symptoms, family history, behavioral changes, and physical examination findings. 3) CLINICAL REASONING: "Ataxia", or unsteadiness, affecting the girl could be due to cerebellar involvement most likely resulting from a lesion such as a tumor. The progressive worsening of the symptoms and finding of papilledema, a sign of increased intracranial pressure, may support this. However, drug-related ataxia can also cause progressively worsening gait disturbance, and the presence of marijuana and cocaine metabolites in her urine makes this a possible consideration. A brief loss of consciousness could point towards an incidence of head trauma leading to formation of an epidural hematoma. Yet, this would likely cause acute and severe symptoms, inconsistent with her 2-month progressive course. Juvenile-onset parkinsonism and multiple sclerosis are neurodegenerative conditions which could potentially lead to gait disturbances. However, their early stages typically do not manifest with such severe and progressive ataxia. Also, it's worth noting the presence of wide-based gait and the inability to perform tandem walk which are characteristic symptoms of cerebellar disturbances rather than parkinsonism or multiple sclerosis. 4) CONCLUDING: From careful gradual analysis of the patient's features, ruling out other options based on reasons as stated previously, it is safe to formulate that the patient's wide-based gait and inability to perform tandem walk are the consequence of a cerebellar dysfunction which could be a result of a cerebellar tumor (Option A). 5) RESPONSE: The most plausible initial diagnosis for this 15-year-old girl's progressively worsening gait disturbance, is a Cerebellar tumor (Option A).

**AI_C:** RESTATING THE QUESTION: A 15-year-old girl presents with behavioral changes, gait disturbance, and frequent falls for the past 2 months. Her medical history includes asthma treated with corticosteroids. There is a significant family history, with multiple sclerosis in a maternal aunt, parkinsonism in her paternal grandfather, and cancer including hemangioblastoma in several paternal relatives. While her vital signs are normal, she displays anti-social behaviour, has expiratory wheezing on lung auscultation, mild bilateral papilledema, and difficulty with tandem walking. Drug screening is positive for marijuana and cocaine. We need to identify the most probable initial diagnosis from the possible choices of cerebellar tumor, drug-related ataxia, epidural hematoma, juvenile-onset parkinsonism, and multiple sclerosis. PLANNING: The significant findings point toward a neurological condition, given her gait disturbances, bilateral papilledema, behavioral changes, and family history. However, some of these findings could also be explained by drug abuse. Therefore, two major alternative assumptions that arise are: a neurological disorder irrespective of the drug use; and a neurological effect due to drug use. The absence of severe head-trauma symptoms suggests that her brief loss of consciousness might not be due to an acute condition like epidural hematoma. Her age and symptoms could align with juvenile-onset parkinsonism, but this is usually a genetically inherited condition and the family history does not specifically support this. Given the longitudinal progression of her symptoms, multiple sclerosis could be a valid consideration. Cerebellar tumor fits most closely with the given findings unless drug use impacts the symptom profile distinctly. CLINICAL REASONING: Focusing on a neurological approach, the bilateral papilledema suggests increased intracranial pressure, which often occurs in space-occupying lesions like a cerebellar tumor. The gait disturbance, behavioral changes, and falls could be signs of cerebellar dysfunction, contributing to the hypothesis of a cerebellar tumor. While family history suggests a genetic predisposition for neurological diseases, none of the family diseases correspond well with the presentation of the patient. On the other hand, drug-related ataxia would typically resolve in the absence of the offending agents and does not usually cause papilledema. CONCLUDING: Connecting the clinical reasoning step-by-step, her presentation consisting of progressively worsening gait disturbance, behavioral changes, bilateral papilledema, and the absence of other focal neurological signs, the most likely diagnosis would be a cerebellar tumor. While the drug use is concerning, it is unlikely to cause all of the symptoms, especially the papilledema. RESPONSE: The teenager's symptoms indicate a cerebellar tumor (option A) as the most likely initial working diagnosis, which should be investigated further clinically and radiologically for confirmation.

**AI_A:** RESTATING THE QUESTION: A young lady, aged 15 has been brought to the clinic by her parents due to the progressive deterioration of her ability to walk over the past two months. She has fallen multiple times and experienced a brief period of unconsciousness following a head injury. Her parents report behavioral changes from the norm. Her existing medical history includes treatable asthma via an as-needed corticosteroid inhaler. Both a maternal aunt and paternal grandfather have nervous system disorders (multiple sclerosis and parkinsonism), and her paternal relatives have a history of cancer. The patient's vital signs are within normal limits. She's conscious and alert, although she avoids eye contact and does not respond to inquiries. Her lung examination reveals expiratory wheezing, indicative of her asthma but other physical examinations produced normal findings. However, mild papilledema is visible in her eyes, indicating the presence of increased intracranial pressure. She exhibits instability in her walk and stance and is unable to execute a tandem walk successfully. Her toxicology results revealed marijuana and cocaine metabolites within her system. The question requires us to ascertain the initial working diagnosis in light of the provided details. PLANNING: The symptoms present in the patient suggest several possible diagnosis. The progressive gait disturbance, avoidance of eye contact and reluctance to communicate could suggest a neurological condition such as a cerebellar tumor (A) or multiple sclerosis (E). The family history of neurological diseases, like multiple sclerosis in the maternal aunt and parkinsonism in her paternal grandfather, strengthen the assumption of a neurological condition. Additionally, mild bilateral papilledema (optic nerve swelling due to increased intracranial pressure) also favors a diagnosis related to the central nervous system. However, positive toxicology for marijuana and cocaine metabolites introduces an alternate assumption of drug-related ataxia (B) as these substances may cause various neurological symptoms. CLINICAL REASONING: The wide-based stance and gait, combined with inability to perform a tandem walk, indicate a problem with the patient's balance and coordination, raising suspicion of a cerebellar issue. Behavioral changes can be indicative of both a neurological condition or substance misuse. Papilledema, present in the patient, is frequently seen with diseases causing increased intracranial pressure like a tumor; the history of cancer on her paternal side gives some weight to this possibility. However, papilledema can also be due to other conditions that increase intracranial pressure, such as high altitude or certain drugs. The positive toxicology helps tie the behavioral changes and neurological symptoms to substance use, opportunely highlighted by the presence of cocaine, which is known to produce such neurological and psychiatric manifestations. CONCLUDING: Given the increasingly worsened walking pattern over two months, the abnormal balance and coordination coupled with behavioral changes and positive toxicology report, it's plausible these are neurological effects due to the substance misuse. Despite the family history of nervous system disorders, the presence of drug metabolites swings our judgement towards a diagnosis of drug-related ataxia, as these substances are well-documented in the literature for having significant neurologic effects. Moreover, the temporal nature of the symptoms (2 months) aligns with a more sudden intervention such as drug use, rather than slow and progressive pathologies like tumors or neurodegenerative disorders. RESPONSE: Therefore, based on clinical reasoning and evaluating the patient's symptoms and history, the most likely initial working diagnosis would be (B) Drug-related ataxia.

**AI_D:** RESTATING THE QUESTION: A young girl has been brought in by her parents who present with a 2-month history of problems with her walking, which they have explained is getting worse, making her fall and injure herself. These problems have also been linked to changes in her behavior. She has a known condition of asthma and has been using a corticosteroid inhaler for treatment. Upon examination, some notable findings include: mild bilateral papilledema, she has a wide posture and shows difficulty in walking straight. Her urine screening test showed presence of marijuana and cocaine. Also, it is noteworthy that her family members have diseases such as multiple sclerosis, parkinsonism, and cancer, specifically, a grandmother who died from a hemangioblastoma. The question now is, considering her symptoms, examination findings, her medical and family history, and drug screening results, what is the most probable initial diagnosis? PLANNING: The assumptions here include the relationship between her symptoms and family history, the effect of her drug use, and whether her symptoms are a result of her current medication for asthma. Alternate assumptions to consider may include the possibility of brain-related disorders such as multiple sclerosis, parkinsonism, or a tumor, given her symptoms and family history, or the potential effects of her drug use on her condition. Focusing specifically on her gait disturbance, behavioral changes, the presence of bilateral mild papilledema, her parents' observation of her behavior, and her inability to perform a tandem walk, a neurological issue is likely to be the most clinically significant. CLINICAL REASONING: The presence of papilledema, which is swelling of the optic disc due to increased intracranial pressure, could be a sign of a brain tumor resulting in her gait disturbances. This is supported by her family's history of cancer, particularly her grandmother's death from a hemangioblastoma, a tumor of the central nervous system. While her urine toxicology screen is positive, the symptoms she presents have been progressive over two months, which points to a more chronic condition not typically associated with acute drug intoxication. Furthermore, her family history of multiple sclerosis and Parkinson's disease could lead one to consider them as possibilities. However, multiple sclerosis does not typically present with papilledema and juvenile Parkinson's is very rare. CONCLUDING: Taking an in-depth look into her history, her progressive symptoms - namely gait disturbance, changes in behavior, papilledema - as well as her family history of neurological disorders and tumors, leads to the possibility of a neurological condition. The working diagnosis should be supported by the chronicity and presentation of her symptoms, her examination findings (such as papilledema, which points to increased intracranial pressure), as well as the prevalence of such a condition, especially when considering her age. RESPONSE: Given the progressive nature of the symptoms, the results of the physical examination, positive family history of central nervous system tumors, in the presence of papilledema which signifies increased intracranial pressure, the most likely initial working diagnosis for this case would be (A) Cerebellar tumor. This condition would bring about disturbances in her gait and potentially behavioral changes, and it aligns closely with her presented symptoms. Additionally, her family history of tumors further supports this diagnosis.

**Facilitator** **[Round 2]:** Summary: AI_A believes the most likely diagnosis is drug-related ataxia, based on the positive toxicology report and the temporal nature of the symptoms. AI_B, AI_C, AI_D, and AI_E all believe the most likely diagnosis is a cerebellar tumor, based on the presence of papilledema, the progressive nature of the symptoms, and the family history of nervous system disorders and cancer. AI_B, AI_C, and AI_D also rule out other conditions such as multiple sclerosis, juvenile-onset parkinsonism, and epidural hematoma based on the patient's symptoms and family history. Key Differences: The key difference in the perspectives lies in the weight each AI gives to the positive toxicology report. AI_A believes the presence of drug metabolites is the most significant factor, leading to a diagnosis of drug-related ataxia. The other AIs, however, believe the presence of papilledema, the progressive nature of the symptoms, and the family history of nervous system disorders and cancer are more indicative of a cerebellar tumor. Question for Council Members: How do you weigh the significance of the positive toxicology report against the other symptoms and family history in determining the most likely diagnosis?. Based on your preceding response, how would you answer the original question: [A 15-year-old girl is brought to the office by her parents because of a 2-month history of progressively worsening gait disturbance. She has fallen several times, once striking her head and causing a brief loss of consciousness. Her parents state that she has had behavioral changes during this time. Medical history is remarkable for asthma treated with a corticosteroid inhaler as needed. Family history is remarkable for multiple sclerosis in a maternal aunt, parkinsonism in her paternal grandfather, and cancer in several paternal relatives, including a grandmother who died from a hemangioblastoma. Vital signs are normal. The patient is awake and alert, but she avoids eye contact and will not answer questions. Auscultation of the lungs discloses expiratory wheezing. Cardiac and abdominal examinations disclose no abnormalities. She has mild bilateral papilledema. Her stance and gait are wide-based, and she is unable to perform a tandem walk. Toxicology screening of the urine is positive for marijuana and cocaine metabolites. Which of the following is the most likely initial working diagnosis? (A) Cerebellar tumor (B) Drug-related ataxia (C) Epidural hematoma (D) Juvenile-onset parkinsonism (E) Multiple sclerosis]

**AI_A:** Restating the original question: Taking into account this 15-year-old girl's clinical presentation, including her progressively worsening gait disturbance, avoidance of eye contact, refusal to answer questions, and positive toxicology screening for marijuana and cocaine along with a family history of multiple sclerosis, parkinsonism, and cancer, we're asked to determine the most probable initial diagnosis. Planning: At this juncture, three critical assumptions are worth considering: cerebellar tumor, drug-related ataxia, or a neurological disease such as Multiple Sclerosis (MS) or Juvenile-onset Parkinsonism based on the family history. We can analyze each assumption by looking at the patient's reported symptoms and family history. The priority would be to consider the most life-threatening diagnoses, such as a cerebellar tumor. Clinical Reasoning: The progressive nature of the symptoms, the patient's wide-based gait, and inability to tandem walk suggest cerebellar dysfunction. Positive toxicology screens indicate substance use, but they do not definitively imply that the patient's symptoms derive solely from substance consumption. Bilateral papilledema could indicate increased intracranial pressure, a sign often seen in brain tumors. Although there is a familial history of MS and Parkinsonism, this patient's symptoms do not align neatly with those diseases' typical presentations. Concluding: After a thorough analysis of the current clinical picture juxtaposed with the patient's family's medical history and the patient's toxicology screening, the most probable initial diagnosis is a cerebellar tumor. Response: Thus, given the signs of cerebellar dysfunction and the presence of bilateral papilledema indicating possible increased intracranial pressure, the most likely initial working diagnosis, in this case, would be (A) a cerebellar tumor.

**AI_B:** RESTATING THE QUESTION: A 15-year-old girl is presented with a 2-month history of progressively worsening gait disturbance, behavioral changes and a few incidents of falling. She's additionally displaying symptoms of avoidance in social interaction and mild bilateral papilledema. The family history is notable for multiple sclerosis, parkinsonism and cancer. Her medical history includes asthma and recent toxicology reports show the presence of marijuana and cocaine metabolites. We need to identify the most probable initial working diagnosis from five options: Cerebellar tumor, Drug-related ataxia, Epidural hematoma, Juvenile-onset parkinsonism, or multiple sclerosis. PLANNING: We know that cerebellar tumors can cause symptoms like gait disturbances and papilledema. Drug-related ataxia could also manifest with gait disturbances and behavioral changes, but it typically wouldn't explain papilledema. An epidural hematoma could theoretically account for these symptoms, but this is less likely given the gradual progression of symptoms. Juvenile-onset parkinsonism and multiple sclerosis usually don't present with papilledema either. CLINICAL REASONING: Starting with drug-related ataxia, while the positive toxicology screen suggests recent drug use this would typically cause acute changes rather than a progressive two-month history. Epidural hematoma generally presents with acute symptoms following head trauma, not a slow progression of symptoms over months. The presentation of Juvenile-onset parkinsonism usually includes tremors, bradykinesia and postural instability, which are not evident here. Multiple sclerosis, typically presents with symptoms that come and go, rather than the progressive worsening seen in this case. This brings us back to a cerebellar tumor. The presence of Papilledema suggests a disorder affecting the central nervous system and causing increased intracranial pressure. The gradual deterioration of her gait and change in behavior, in conjunction with papilledema and a history of similar cerebellar tumor in the family makes this the most likely initial working diagnosis. CONCLUDING: From clinical reasoning, the progressive deterioration of gait, changes in behavior associated with papilledema and family history of cerebellar tumor, seems to outweight the effects of recent drug usage and asthma. RESPONSE: Therefore, in answering the posed question, '(A) Cerebellar tumor' is the most likely initial working diagnosis among given options with the provided context and symptoms. This diagnosis aligns with the majority consensus within the council. However, definitive diagnostic confirmation would necessitate further imaging evaluations.

**AI_E:** Restating the question: The question is asking what the initial diagnosis may be for a 15-year-old female patient showing progressive gait disturbance, behavioral changes, and positive toxicology screenings for marijuana and cocaine use. She also displays some family history of neurological disorders and cancer. Planning: Several assumptions can be derived from the question: 1) Assumption 1: The patient's symptoms are primarily neurologic, with the presence of a wide-based stance, gait disturbance, and mild bilateral papilledema all suggesting potential CNS involvement. 2) Assumption 2: The patient's symptoms could be related to her drug use, as drug-related ataxia can result from cocaine or marijuana metabolite presence. 3) Assumption 3: The family history of multiple sclerosis, parkinsonism, and cancer may signify genetic predispositions. For the clinical evaluation, we should focus on Assumption 1 because the key presentations (papilledema and gait disturbance) can't be explained solely by drug use or family history. Clinical Reasoning: - The clinical presentation makes clear that there are neurological indications (gait disturbance, papilledema). - Cocaine and marijuana use can lead to myriad neurological effects, but these effects are usually transit, unlike the persistent, progressively worsening signs in this case. - Although there's a significant family history of both neurological disorders (multiple sclerosis, parkinsonism) and cancer, the patient's age and the abrupt, progressive nature of the symptoms suggest an underlying condition that's urgent (like a cerebellar tumor). Therefore, a drug-related ataxia, multiple sclerosis or juvenile-onset parkinsonism seem unlikely. An epidural hematoma would most likely present more acutely after head trauma. Concluding: The progressive nature of the symptoms, the positive tox screening for marijuana and cocaine, the family history of cancer, and the presence of papilledema are all significant factors. While each can contribute to the overall health picture, taken together, they suggest a serious, ongoing issue. Response: Given these factors, the most likely initial working diagnosis is a cerebellar tumor (Option A). The severe progressive symptoms and subtle signs of increased intracranial pressure (papilledema) hint towards a developing mass lesion, such as a cerebellar tumor. Further investigation through neuroimaging would be essential to confirm this diagnosis.

**AI_C:** Restating the Question: The inquiry at hand revolves around a 15-year-old girl who has been experiencing a progressively worsening gait disturbance culminating in falls, one that led to a brief loss of consciousness. Her parents have noticed behavioral changes. She has asthma managed with a corticosteroid inhaler. Significant family history exists of multiple sclerosis, parkinsonism, and cancer, including a hemangioblastoma. On examination, she has a wide-based stance and is unable to carry out the tandem walk test. A toxicology screen revealed the presence of marijuana and cocaine metabolites. The question demands an initial working diagnosis from the following options: a cerebellar tumor, drug-related ataxia, an epidural hematoma, juvenile-onset parkinsonism, or multiple sclerosis. Planning: To ascertain the likeliest diagnosis, it’s crucial to analyze all observable symptoms and her family medical history. The initial concern that springs to mind is a drug-related ataxia, given the presence of drug metabolites in her system. However, her complex medical history and the observed symptoms could also indicate a potentially more severe neurological condition, possibly a cerebellar tumor. Clinical Reasoning: Toxicology results revealed the presence of marijuana and cocaine metabolites, substances known to interfere with motor skills or cognitive functions, which could possibly explain her gait disturbances and behavior changes. However, these abnormalities shouldn’t induce papilledema –a clear symptom of increased intracranial pressure– unless they have caused severe cerebral edema or, less likely, an epidural hematoma which is discounted since the onset wasn’t sudden or severe enough. The sustained and degenerative symptomatology, combined with the papilledema, roughens the endeavor of associating these symptoms with drugs solely. The other disorders: MS, juvenile-onset parkinsonism, are less likely in light of her symptoms and family history. MS generally doesn’t cause papilledema or lead to such quick, aggressive symptom deterioration. Juvenile-onset parkinsonism doesn’t typically present until late adolescence or early twenties. The positive family history of nervous system disorders and cancer, especially hemangioblastoma, should not be underestimated. Given the evidence, our assertion should lean towards a grave neurological condition such as a cerebellar tumor. This conclusion is inferred from the patient’s progressive extremity incoordination, gait disturbance, behavioral changes, family history of nervous system maladies, and presence of papilledema. Concluding: Thus, I agree with AIs B, C, D, and E that the initial working diagnosis that should be prioritized for further investigation, based on the available evidence and clinical reasoning, is a cerebellar tumor (A). Response: With the clinical background and reasoning stated above in mind, I would respond to the question by stating that the most likely initial working diagnosis is (A) Cerebellar tumor.

**AI_D:** RESTATING THE QUESTION: The question presents the case of a 15-year old girl with symptoms such as a progressive gait disturbance, fall injuries, behavioural changes, and avoidance of eye contact. Notably, she has slight bilateral papilledema, a wide-based stance and gait, and can't do a tandem walk. Even though her toxicology screening tests positive for marijuana and cocaine, her significant family history related to multiple sclerosis, parkinsonism, and cancer, including hemangioblastoma, is noteworthy. The question wants to distinguish between five possible diagnoses: cerebellar tumor, drug-related ataxia, epidural hematoma, juvenile-onset parkinsonism, or multiple sclerosis. It's important to define some of these terms: papilledema refers to optic disc swelling due to increased intracranial pressure; a wide-based gait could indicate a problem with proprioception or balance; a tandem walk test can reveal impaired coordination (ataxia); ataxia refers to a lack of muscle control during voluntary movements; an epidural hematoma is bleeding between the skull and the dura mater. PLANNING: There are several assumptions involved here: (1) the symptoms have been caused by drug use, (2) her family history of neurological diseases is relevant, and (3) the findings of papilledema imply raised intracranial pressure. By questioning each assumption, we could consider the possibility that her symptoms may have resulted from a neurological condition distinct from her drug use or family history. However, given the progressive nature of her symptoms, her family history, and the papilledema, it seems clinically sound to prioritize exploring the hypothesis concerning a cerebellar tumor, which may plausibly account for all these factors. CLINICAL REASONING: Progressive gait disturbance could be a sign of a developing neurological condition, rather than an episodic influence from drug use. Papilledema is typically associated with conditions that increase intracranial pressure, such as a tumor. Her wide-based gait and inability to perform a tandem walk may indicate cerebellar dysfunction, which is consistent with a possible cerebellar tumor. Moreover, considering her significant family history of cancer, including a relative with a brain tumor (hemangioblastoma), it would make sense to investigate a cerebellar tumor as a potential cause. CONCLUDING: Based on her presenting symptoms, the progression over two months, the presence of papilledema, her gait difficulties, the family history of nervous system disorders and cancer, and ruling out others - particularly drug related ataxia which would be episodic in nature - my final conclusion is that the most likely initial working diagnosis for this patient would be a cerebellar tumor. RESPONSE: From connecting the factors and evaluating the clinically prioritized assumptions, my answer to the original question would be: (A) Cerebellar tumor.

**Facilitator** Restating the question: The case involves a 15-year-old girl with a 2-month history of progressively worsening gait disturbance, behavioral changes, and positive toxicology screenings for marijuana and cocaine. She also has a family history of multiple sclerosis, parkinsonism, and cancer. The question asks for the most likely initial working diagnosis. Planning: The assumptions to consider include the possibility of a cerebellar tumor, drug-related ataxia, or a neurological disease such as Multiple Sclerosis (MS) or Juvenile-onset Parkinsonism based on the family history. The priority is to consider the most life-threatening diagnoses, such as a cerebellar tumor. Clinical Reasoning: The progressive nature of the symptoms, the patient's wide-based gait, and inability to tandem walk suggest cerebellar dysfunction. Positive toxicology screens indicate substance use, but they do not definitively imply that the patient's symptoms derive solely from substance consumption. Bilateral papilledema could indicate increased intracranial pressure, a sign often seen in brain tumors. Although there is a familial history of MS and Parkinsonism, this patient's symptoms do not align neatly with those diseases' typical presentations. Concluding: After a thorough analysis of the current clinical picture juxtaposed with the patient's family's medical history and the patient's toxicology screening, the most probable initial diagnosis is a cerebellar tumor. This diagnosis aligns with the majority consensus within the council. However, definitive diagnostic confirmation would necessitate further imaging evaluations. Response: Thus, given the signs of cerebellar dysfunction and the presence of bilateral papilledema indicating possible increased intracranial pressure, the most likely initial working diagnosis, in this case, would be (A) a cerebellar tumor.
